# At-Point Clinical Frailty Scale as a Universal Risk Tool for Older Inpatients in Acute Hospital: A Cohort Study

**DOI:** 10.3389/fmed.2022.929555

**Published:** 2022-07-06

**Authors:** Hee-Won Jung, Ji Yeon Baek, Young hye Kwon, Il-Young Jang, Dae Yul Kim, Hyouk-Soo Kwon, Sun hee Lee, Hyun jin Oh, Eunju Lee, Younsuck Koh

**Affiliations:** ^1^Division of Geriatrics, Department of Internal Medicine, Asan Medical Center, University of Ulsan College of Medicine, Seoul, South Korea; ^2^Department of Nursing, Asan Medical Center, Seoul, South Korea; ^3^Department of Rehabilitation Medicine, Asan Medical Center, University of Ulsan College of Medicine, Seoul, South Korea; ^4^Department of Internal Medicine, Department of Allergy and Clinical Immunology, Asan Medical Center, University of Ulsan College of Medicine, Seoul, South Korea; ^5^Department of Pulmonary and Critical Care Medicine, Health Screening and Promotion Center, Asan Medical Center, Seoul, South Korea

**Keywords:** Clinical Frailty Scale, adverse health outcomes, in-hospital outcomes, screening tool, older adults

## Abstract

**Background:**

While the Clinical Frailty Scale (CFS) has been extensively validated for predicting health outcomes in older adults, the role of the at-point CFS at the time of examination is unclear. We aimed to examine the ability of the at-point CFS for predicting clinical outcomes of older inpatients.

**Methods:**

As a single-center and prospective cohort study, we enrolled 1,016 older adults who were 65 years or older and were admitted to one of 9 medical or surgical units from May 2021 to September 2021. The associations of the at-point CFS with outcomes of falls, delirium, pressure ulcers, 30-day unplanned readmission and/or emergency department (ED) visits, institutionalization, and a composite outcome were analyzed.

**Results:**

In the study population (*n* = 1,016), 26 patients had incident pressure ulcers, 6 patients had falls, 50 patients experienced delirium, and 13 patients died during hospitalization. Also, 37 patients experienced an ED visit and 22 patients had an unplanned readmission within 30 days after discharge. The composite outcome was 1.7% among patients with the CFS < 5 and 28.5% among patients with the CFS ≥ 5. The higher CFS was associated with an increased risk of a fall [odds ratio (OR) 1.74 (1.01–3.01)], pressure ulcers [OR 3.02 (2.15–4.23)], delirium [OR 2.72 (2.13–3.46)], 30-day readmission [OR 1.94 (1.44–2.62)], ED visit [OR 1.81 (1.47–2.23)], death [OR 3.27 (2.02–5.29)], and institutionalization after discharge [OR 1.88 (1.62–2.18)].

**Conclusion:**

The at-point CFS assessed in older inpatients can screen high-risk individuals who might experience adverse geriatric conditions and in-hospital outcomes.

## Introduction

Frailty is an age-related condition that is defined as a state of decreased physiological reserve and increased vulnerability to adverse outcomes due to the accumulation of biological aging processes (1, 2). While initially discovered and studied as a clinical construct (3), subsequent research has validated frailty as a biologically relevant measure of aging in humans and animal models, such as mice, rats, and nonhuman primates (4–6). Clinical studies have shown the outcome relevance of frailty in diverse medical or surgical conditions (7, 8) and the clinical consequences of frailty, namely, immobility, functional decline, falls, and cognitive impairment, could be managed and alleviated by appropriately designed interventions (9). There have been numerous frailty assessment tools; however, two types of frailty measurements are dominant: frailty phenotype and frailty index (4). Frailty phenotype emphasizes the importance of physical decline, including five clinical parameters: unintended weight loss, weakness, low physical activity, slow walking speed, and exhaustion. The total counted number of applicable parameters, ranging from 0 to 5, is determined as a frailty score (3). The other concept of defining frailty is the frailty index, which calculates the proportion of deficits among more than 30 age-related parameters having an association with adverse health outcomes (10).

Many older individuals experience hospitalizations as their burden of clinical diseases and subclinical pathologies accumulate with aging. In acute clinical situations, older patients with frailty are more likely to suffer from geriatric syndromes such as delirium and adverse health outcomes after discharge as compared with individuals without frailty (11–13). To minimize adverse clinical outcomes while preserving the functional status of older patients, models of geriatric acute care have been developed and shown to have clinical benefits (14). Also, screening measures of frailty have been studied to identify vulnerable populations who might benefit from person-centered, geriatric-focused care provisions (15–17).

Among many tools for measuring frailty, the Clinical Frailty Scale (CFS) (18), a scale ranging from 1 to 9 with descriptions and pictograms, has been widely used in various clinical settings from the emergency department (ED) to chronic care facilities as a measure of the degree of frailty (13, 18, 19). As the tool summarizes key functional features of a Comprehensive Geriatric Assessment, numerous studies support its construct validity and the clinical relevance of the CFS in aged populations (13). With its simplicity and strong prediction ability for health outcomes, the CFS has been even advocated as a potential triage tool to make decisions such as allocating scarce healthcare resources in case surges of the COVID-19 pandemic in some countries (20), and a prognostic indicator *per se* (21, 22).

In acute medical situations, the functional status may change significantly in a short time. Hence, the CFS is supposed to be applied to assessing the baseline functional status by asking how the person performed 2 weeks ago, before the person became acutely ill (20, 23). In prognosticating patients, a recent article by Rockwood and Theou suggested that the CFS could be used to assess acute severity (20). Given at-point the CFS includes not only baseline functional status, but also an acute decline of inpatients, it is expected to evaluate the health status of patients at the time of presentation. However, the clinical implications of the at-point CFS have not been reported. In acute hospitalization, we hypothesized that the CFS at admission may serve as a measure of the vital sign of older adults and have role in identifying high-risk patients who may experience adverse health outcomes during and after the index hospitalization.

## Methods

### Study Design, Setting, and Population

This study was a prospective cohort study from Asan Medical Center, a tertiary teaching hospital in Seoul, Korea. From May 2021, a multidisciplinary team started to measure the CFS in 9 acute inpatient units encompassing 24 medical and surgical specialties/subspecialties, as one part of our activities to develop an age-friendly health system, which was proposed by The John A Hartford Foundation and the Institute for Healthcare Improvement (IHI) for establishing evidence-based high-quality care for older adults throughout the hospital. All the older inpatients aged 65 years or older in these 9 units were screened by a geriatric nurse specialist. A geriatric nurse visited these units and measured the CFS and patient-specific needs in domains of “What matters,” “Medication,” “Mentation,” and “Mobility,” as part of a process of developing care pathways to link these needs with resources from Monday to Friday (8 a.m. to 6 p.m.). For these assessments, we included patients admitted through both the outpatient clinic and the ED and the geriatric nurse specialist visited patients on the morning of the day after admission. We included patients 65 years or older admitted to these units from May to September 2021 for this study. The exclusion criteria were patients who needed to be quarantined because of infection issues, received radiation therapy within 24 h, and went through discharge within 24 h. As all the measures were conducted by a single nurse, patients admitted on Saturday were also excluded. The average number of enrolled inpatients in a day was about 15–20. This study protocol was reviewed and approved by the Institutional Review Board of Asan Medical Center (Approval Number: 2021-1523). The written informed consent was waived, as evaluating the general health status of patients at admission is a routine procedure, and no additional harm was expected. To best reflect the real-world settings embracing a wide range of clinical circumstances, as we are aiming to develop an acute electronic frailty pathway, including the entire hospital, there were no exclusion criteria for applying the CFS.

### At-Point the Clinical Frailty Scale

The CFS was measured once on the day afterward admission by a trained geriatric nurse specialist who had completed a 2-year geriatric nurse specialist course and >10 years of experience in clinical units of rehabilitation medicine, neurology, and geriatrics, participated in the process of Korean translation, and adoption of the CFS 2.0 in the institution. We used the Korean-translated version of the CFS 2.0 that has had its construct validity established in Korean geriatric outpatients (24) and its accuracy for predicting adverse outcomes in hospitalized older medical patients was demonstrated (12). For consistent scoring of the CFS, we measured the CFS by using the classification tree (25). Patients' activity of daily living, the instrumental activity of daily living, and self-rated health status were investigated by a geriatric nurse. While the original CFS was intended to be used to assess the baseline functional status before acute medical deterioration, we used the at-point CFS, which combines both the baseline functional status and acute deterioration and presents the current functional state of the persons in this study. From prior observations, we considered the CFS scores ≥ 5 to indicate frailty (12, 13). For patients who are unable to communicate due to altered mental status or cognitive problems, functional status was assessed by interviewing their direct caregivers in person or over the phone.

### Clinical Parameters

Demographic factors and the pathway of admission (ED vs. outpatient clinics) were recorded. Vital signs on the morning of the day of the assessments were recorded. From the medical records, clinical diagnoses of angina, arthritis, asthma, cancer, chronic lung disease, congestive heart failure, dementia, depression, diabetes, myocardial infarction, hypertension, chronic kidney disease, spine problems, and stroke were reviewed. The risk of falls and pressure ulcers were assessed by the Morse Fall Scale (MFS) and the Braden Sore Scale (BSS) that were applied by the nursing staff on the day of examination (26, 27). For clinical laboratory parameters, we used hemoglobin and serum albumin levels taken at the admission date.

### Outcome Measures

By medical record review, we assessed the incidence of new pressure ulcers, delirium, and in-hospital death. Delirium was detected by medical review, as the presence of either clinical remark, nursing diagnosis, or consultation to psychiatry or geriatrics due to clinical suspicion of delirium. Fall incidence was acquired from the fall reports that are mandatory for every fall event throughout the hospital. Length of stay (LOS) was recorded. We defined a long hospital stay as a LOS of 14 days or longer. Unplanned ED visits and readmission within 30 days after discharge were reviewed. The location of discharge was assessed by medical review. We defined a composite outcome, including events of new bed sores, delirium, falls, in-hospital death, 30-day unplanned readmission, or an ED visit.

### Statistical Analysis

For sample size, we used a previous report using the CFS in older inpatients of a Korean acute medical unit (12) that reported in-hospital mortality of 1.3 and 4.6% in patients with the CFS < 5 (54.9% of total population) and the CFS ≥ 5 (45.1% of total population), respectively. With alpha of 0.05 and beta of 0.80, the total sample size of 814 was required. We aimed to collect records of 1,016 patients, with a safe margin of 20%. We used the *t*-tests for continuous variables and the chi-square tests or the Fisher's exact test for categorical variables to compare clinical characteristics between individuals with the CFS < 5 and the CFS ≥ 5. To assess the correlation of the CFS with the MFS and the BSS, we used a 95% CI fractional polynomial plot for visualization and linear regression analysis with the calculation of the standardized beta (B). The association between the CFS and dichotomized outcomes, namely, falls, new pressure ulcers, delirium, death, length of stay 14 days or longer, 30-day ED visit, readmission, the composite outcome (falls, new pressure ulcers, delirium, death, 30-day ED visit, and readmission), and discharge to a chronic care facility, was assessed by logistic analyses (unadjusted and adjusted models with covariables of age and sex). To evaluate the prediction ability for falls and new pressure ulcers, we performed the receiver operating characteristic (ROC) analyses with the CFS as a classifier and these outcomes as references. Sensitivities and specificities for each CFS score and C-statistics predicting the outcomes were calculated. We considered two-sided *p-*values < 0.05 as statistically significant. Stata version 15.0 was used for the analysis (Stata Corporation, College Station, Texas, USA).

## Results

### Clinical Characteristics

**Figure 1:**
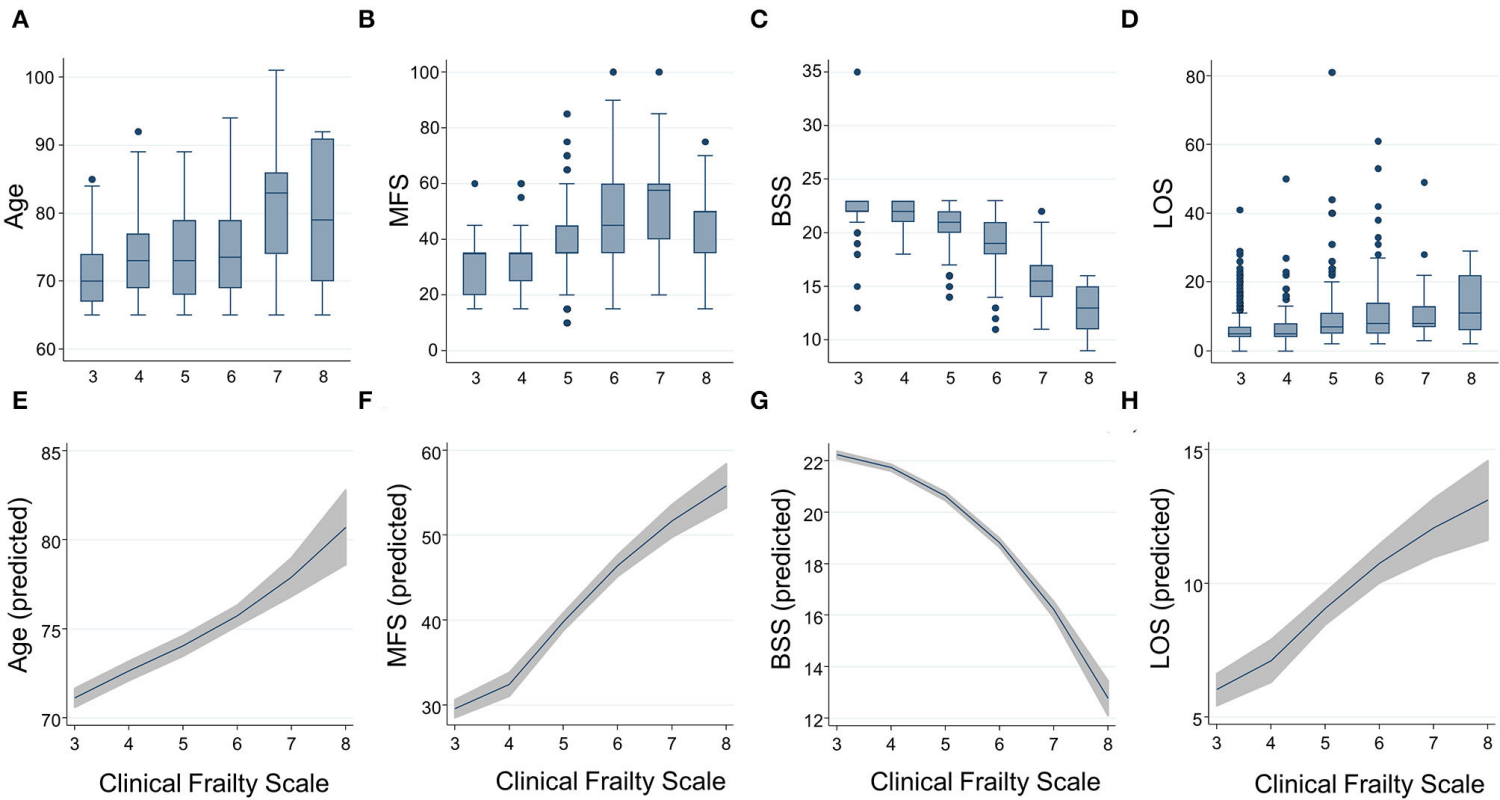
Distributions by box and whisker plots and predicted means by fractional polynomial plots with 95% CIs for age **(A,E)**, the Morse Fall Scale **(B,F)**, the Braden Sore Scale **(C,G)**, and length of stay **(D,H)** according to the Clinical Frailty Scale. BSS, Braden Sore Scale; LOS, length of stay; MFS, Morse Fall Scale.

Among 1,016 patients with a mean age of 73.0 years (SD 6.2) and 415 (40.9%) women, 329 (37.3%) patients had the CFS of 5 or higher. Specifically, 461 (45.4), 176 (17.3), 170 (16.7), 154 (15.2), 44 (4.3), and 11 (1.1%) individuals had the CFS score of 3–8, respectively. The clinical characteristics of patients with the CFS < 5 or ≥ 5 are shown in Table 1. Individuals with the CFS ≥ 5 were older, more likely to be women, admitted through the ED, and had a higher burden of comorbidities such as hypertension or diabetes. Their hemoglobin and albumin levels were lower and experiences of falls in the previous year were higher in the frail population. During the hospitalized period, the probabilities of delirium, pressure ulcers, and falls were all higher in the frail group. Also, the length of stay for the index admission, the likelihood of experiencing an unplanned ED visit, readmission within 30 days after discharge, and in-hospital mortality were higher in the frail group. The CFS correlated with chronological age (*B* = 0.342, *R*^2^ = 0.117, *p* < 0.001, Figures 1A,E) and was higher (*p* < 0.001 by *t*-tests) in women (mean ± 4.48, SD ± 1.34) than that in men (mean ± 3.99, SD ± 1.27).

**Table 1:** Clinical characteristics of the study population.

	**CFS < 5 (*n* = 637)**	**CFS ≥ 5 (*n* = 379)**	***p-*value**
Age (yr)	71.8 ± 5.1	75.0 ± 7.2	<0.001
Women	215 (33.8%)	200 (52.8%)	<0.001
Admitted through ED	20 (3.1%)	128 (33.8%)	<0.001
Surgical departments	404 (63.4%)	180 (47.5%)	<0.001
BMI (kg/m^2^)	24.6 ± 5.6	23.1 ± 3.9	<0.001
Hypertension	301 (47.3%)	223 (58.8%)	<0.001
Diabetes	154 (24.2%)	127 (33.5%)	0.001
Cancer	230 (36.1%)	135 (35.6%)	0.88
Hemoglobin (g/dL)	12.8 ± 1.6	11.4 ± 2.2	<0.001
Albumin (g/dL)	3.9 ± 3.0	3.2 ± 0.7	<0.001
Fall in previous year	48 (7.5%)	109 (28.8%)	<0.001
Incident delirium	1 (0.2%)	49 (13.0%)	<0.001
Incident sore	0 (0.0%)	26 (6.9%)	<0.001
Incident fall	1 (0.2%)	5 (1.3%)	0.03^a^
Length of stay	6.2 ± 4.5	10.4 ± 8.9	<0.001
ED visit in 30 days	9 (1.4%)	38 (10.1%)	<0.001
Unplanned readmission in 30 days	2 (0.3%)	20 (5.3%)	<0.001
In-hospital mortality	1 (0.2%)	12 (3.2%)	<0.001
Composite outcome	11 (1.7%)	108 (28.5%)	<0.001
Discharge to chronic care facilities	39 (6.2%)^b^	75 (20.7%)^b^	<0.001

a *Fisher's exact test*.

b *Data available in 632 individuals with CFS <5 and 363 with CFS ≥5*.

*BMI, body mass index; ED, emergency department*.

### At-Point the Clinical Frailty Scale as a Geriatric Risk Indicator

The CFS correlated with the MFS (*B* = 0.538, *R*^2^ = 0.289, *p* < 0.001) and the BSS (*B* = 0.572, *R*^2^ = 0.328, *p* < 0.001), as the trends shown in Figures 1B,C,F,G. A higher frailty burden estimated by the CFS was associated with increased odds for risks of falls and pressure ulcers as determined by the MFS and BSS (Table 2). C-statistics of the CFS to classify the fall risk (MFS ≥ 45) and pressure ulcer risk (BSS ≤ 18) were 0.884 (95% CI 0.863–0.905) and 0.907 (95% CI 0.880–0.934). A cutoff of the CFS ≥ 5 maximized the sensitivity + specificity of classifying the fall risk (sensitivity 88.9% and specificity 76.6%) and pressure ulcer risk (sensitivity 95.5% and specificity 71.4%). Sensitivities and specificities for the individual CFS scores are shown in Supplementary Table 1.

**Table 2:** Associations between an increasing burden (1 point higher) of frailty by the Clinical Frailty Scale and the risk of geriatric conditions and hospital outcomes by the logistic regression analyses.

	**OR (unadjusted)**	**OR (age, sex-adjusted)**
**Risk of geriatric conditions**		
Fall risk by MFS (MFS ≥45)	3.58 (3.03–4.24)	3.36 (2.83–4.00)
Fall incidence	1.74 (1.01–3.01)	**1.39 (0.74**–**2.60)**
Pressure ulcer risk by BSS (BSS ≤ 18)	5.14 (3.96–6.68)	4.88 (3.74–6.37)
Pressure ulcer incidence	3.02 (2.15–4.23)	2.77 (1.94–3.96)
Delirium incidence	2.72 (2.13–3.46)	2.56 (1.98–3.31)
**Hospital outcomes**		
Length of stay 14 days or longer	1.77 (1.54–2.03)	1.87 (1.61–2.18)
ED visit in 30 days	1.81 (1.47–2.23)	1.96 (1.56–2.45)
Unplanned readmission in 30 days	1.94 (1.44–2.62)	1.99 (1.44–2.76)
In-hospital mortality	3.27 (2.02–5.29)	3.20 (1.94–5.30)
Composite outcome	2.63 (2.22–3.12)	2.54 (2.12–3.03)
Discharge to chronic care facilities	1.88 (1.62–2.18)	1.91 (1.63–2.24)

*BSS, Braden Sore Scale; MFS, Morse Fall Scale; OR, Odds ratio*.

*Statistically insignificant result is highlighted in bold font*.

**Figure 2:**
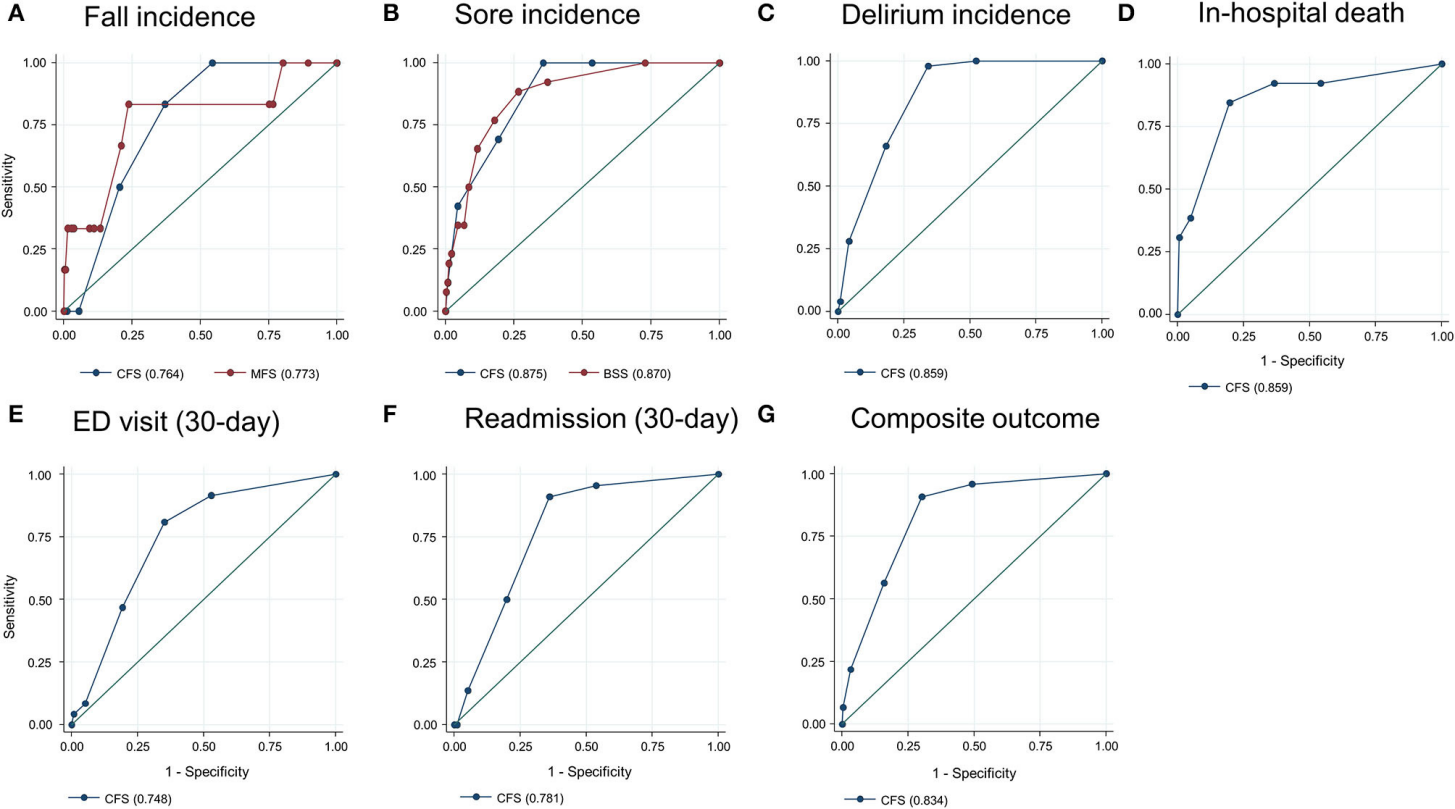
Prediction ability of the Clinical Frailty Scale (CFS) for fall incidence [**(A)**, compared with the Morse Fall Scale (MFS)], sore incidence [**(B)**, compared with the Braden Sore Scale (BSS)], delirium incidence **(C)**, in-hospital mortality **(D)**, 30-day emergency department (ED) visit **(E)**, unplanned 30-day readmission **(F)**, and composite outcome **(G)**. Numbers in parentheses denote the area under the curve.

The total observation time for the incidence of falls and pressure ulcers in this study was 7,885 patients × day. There were 26 incident pressure ulcers, 6 falls (including 1 injurious fall), and 50 patients who experienced delirium during the index hospitalization. A higher frailty burden by the CFS was associated with an increased risk of the incidences of falls, pressure ulcers, and delirium during hospitalization (Table 2), even though the significant association between the CFS and fall incidence was attenuated after adjusting for age and sex. Only 1 patient (CFS 4) below the CFS 5 experienced a fall, which was noninjurious. The prediction ability of the CFS for falls (C-statistic 0.764, 95% CI 0.655–0.872) did not significantly differ from that of the MFS (C-statistic 0.773, 95% CI 0.543–1.000) (Figure 2A). The CFS predicted pressure ulcers incidence (C-statistic 0.885, 95% CI 0.831–0.919) was similar to that of the BSS (C-statistic 0.870, 95% CI 0.811–0.928) (Figure 2B). The CFS was able to predict the incidence of delirium (C-statistic 0.859, 95% CI 0.827–0.892) (Figure 2C). The sensitivities and specificities for the individual CFS scores predicting these outcomes are also shown in Supplementary Table 1.

### Hospital Outcomes

In the study population, 13 patients died during hospitalization, 37 patients experienced an ED visit, and 22 patients had an unplanned readmission within 30 days after discharge. The mean LOS was 7.8 (SD 6.8) days. Patients with the higher CFS tend to stay longer during the index hospitalization (Figures 1D,H), as the CFS was correlated with the LOS (*B* = 0.295, *R*^2^ = 0.087, *p* < 0.001). The higher CFS was associated with a longer hospital stay, 30-day ED visit, unplanned readmission, and in-hospital death either by unadjusted or age- and sex-adjusted logistic regression analyses (Table 2). The CFS was able to predict in-hospital mortality (C-statistic 0.859, 95% CI 0.747–0.972), 30-day ED visit (C-statistic 0.748, 95% CI 0.689–0.806), and unplanned readmission (C-statistic 0.781, 95% CI 0.710–0.852) (Figures 2D–F). As shown in Table 2, the CFS was associated with the composite outcome (falls, new pressure ulcers, delirium, readmission in 30 days, death, and 30-day ED visits) and the C-statistic was 0.834 (95% CI 0.801–0.866) (Figure 2G). Sensitivities and specificities for the individual CFS scores predicting these outcomes are shown in Supplementary Table 2. After excluding individuals deceased or transferred to other acute facilities, 881 individuals were discharged to homes and 114 individuals were discharged to chronic care facilities. The higher CFS scores were associated with institutionalization in chronic care facilities after discharge (Table 2).

## Discussion

In this study, we found that the at-point CFS, capturing the functional state of patients at the time of examination within 24 h after acute admission, could predict both the geriatric outcomes (falls, pressure ulcers, and delirium) and hospital outcomes (death, 30-day ED visit, and 30-day readmission). Also, the prediction ability of the CFS for falls and pressure ulcers was similar to existing scales such as the MFS and the BSS. To the authors' knowledge, this study is the first study to show the potential role of the CFS in acute hospitalization as a measure of geriatric conditions, especially for falls. In contrast to the widespread perception that the CFS should be assessed by evaluating the patient's functional status before the acute deterioration, we observed that the at-point CFS was valid as a risk indicator.

The at-point CFS may detect different clinical constructs of older inpatients than the baseline CFS, which measures global fitness as a reflection of functional deficits accumulated due to aging. In the at-point CFS, baseline functional status and acute deterioration are combined and this measure is sensitive to the rapidly changing clinical course of inpatients in an acute care setting; hence, it is more like a functional vital sign rather than a stable indicator of baseline frailty. Our observation of the good performance of the at-point CFS in predicting various outcomes might be due to this characteristic, as this tool encompasses both the baseline frailty and disease severity. Indeed, our study was in accordance with prior observation with the baseline CFS for inpatients, albeit with a trend of the higher area under the curve value in predicting adverse outcomes even though the direct comparison is not possible considering population characteristics (28). As geriatric conditions such as delirium, falls, and pressure ulcers are consequences of a combination of frailty and disease severity, the at-point CFS might be especially useful as a universal predictor of these outcomes.

Traditionally, the risks of geriatric conditions such as falls, pressure ulcers, and delirium in hospitalized patients have been assessed using tools for the separate conditions (26, 27, 29–31). However, in this prospective population of a large acute hospital, we observed that the at-point CFS could predict various geriatric conditions with no statistically significant differences in prediction ability compared to condition-specific measures such as the MFS or the BSS. Furthermore, the at-point CFS showed construct validity, including convergence validity and criterion validity, for classifying high-risk patients when directly compared with the MFS and the BSS. Based on this evidence and the obvious simplicity of using the CFS when compared to using a combination of the MFS and the BFS, the at-point CFS might be used as a universal measure to screen high-risk populations who may experience an adverse clinical course, including geriatric syndromes.

**Figure 3:**
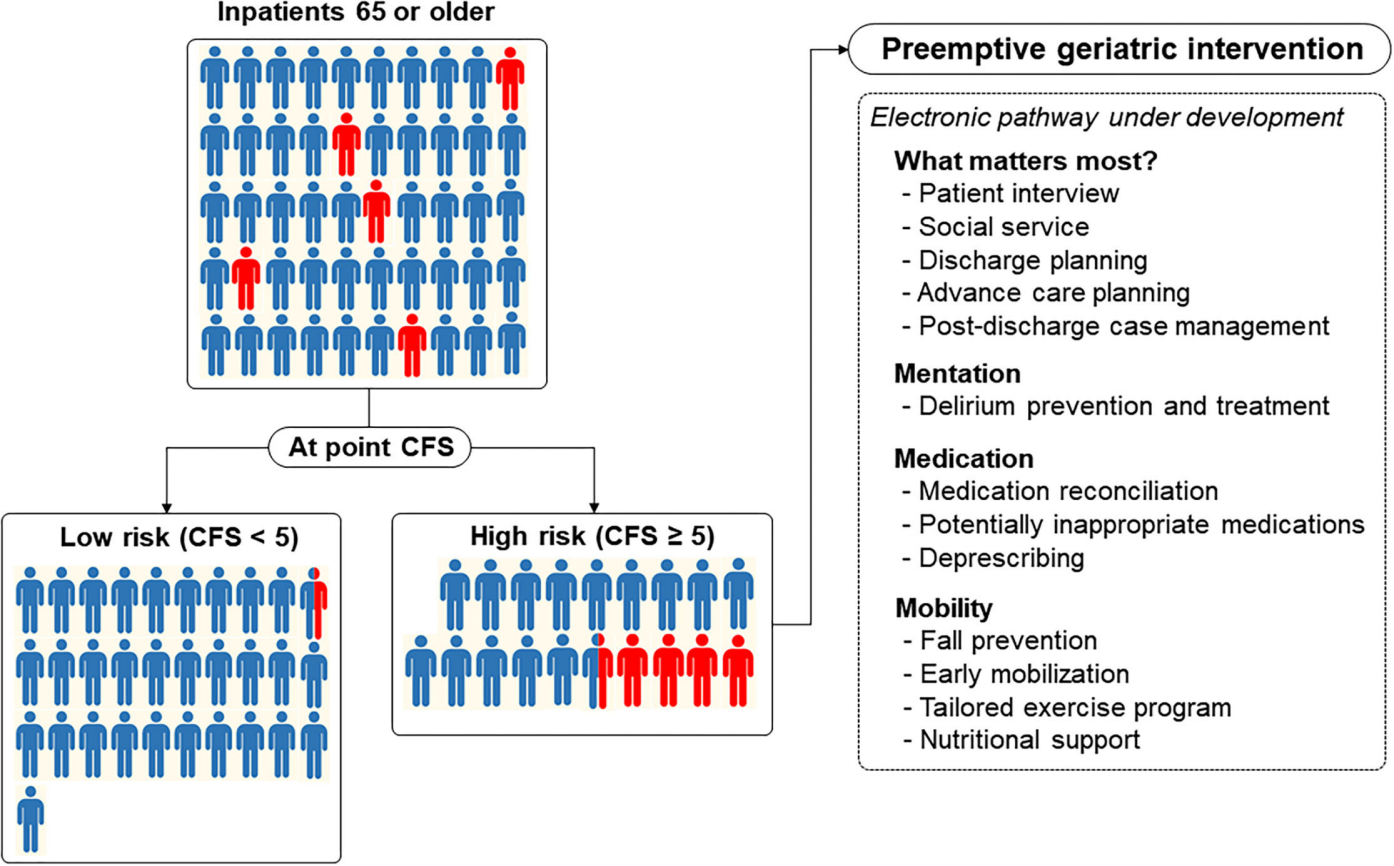
Acute electronic frailty pathway under development using the Clinical Frailty Scale (CFS) as a universal risk indicator for older inpatients to provide a preemptive geriatric intervention encompassing domains of an age-friendly health system. In this figure, a red-colored human denotes a person (1 person denotes 2% incidence) who developed the composite outcome and is shown to reflect the distribution of the CFS and prevalence of the composite outcome in our study.

In large hospitals, performing full geriatric assessment on all the older patients is less feasible. This advantage of the at-point CFS might be leveraged as a case-finding solution for a vulnerable older adult who needs more geriatric attention to prevent adverse outcomes. From this approach, we may maximize both the efficiency and efficacy of adopting the concept of geriatric intervention in a large, acute hospital. One example is our hospital, which is in the process of establishing an acute pathway for older adults by adopting the 4M framework of matter, mentation, medication, and mobility with the Plan-Do-Study-Act (PDSA) cycles (32). As the largest tertiary hospital in Korea with 2,715 beds, one of the most challenging steps in developing a clinical pathway in the hospital would be expanding a standardized way of case finding and care provision while achieving maximal efficiency. In the first step of scaling up with limited resources, screening geriatric risks with separate tools for each condition in all the older inpatients would be less feasible. Based on our findings, we are planning to focus on older adults with the CFS ≥ 5 to provide preemptive, person-centered geriatric interventions on 4M domains through an electronic frailty pathway (Figure 3). To enhance the scalability and efficiency of coordination, pathways embedded in the electronic health records using the CFS measured daily and a 4M framework aiming to provide geriatric care similar to the Acute Care for Elders (ACE) model throughout the hospital are under development (14).

Even though the at-point CFS showed validity in predicting the fall risk in this population, an association between the CFS and fall incidence was attenuated when adjusted for age and sex. This might be potentially affected by the type II error due to the low actual number of fall incidences (6 falls in 7,885 patients × day). Underreporting falls is unlikely, since we use the mandatory hospital administration data that monitors every falls throughout the hospital. The incidence of falls in our data is consistent with prior reports from large hospitals in Korea (33).

There are several limitations to this study. As we did not measure the baseline CFS of patients before their acute clinical conditions, we could not dissect the “acute factor” from the at-point CFS that we measured. Also, as we measured the CFS only once at the baseline, the dynamic nature of the at-point CFS during the clinical course could not be evaluated in this study. Statistical uncertainty might exist as the incidence of delirium that was collected from medical record review and, hence, might be affected by underreporting of hypoactive or mixed delirium. Generalizability is limited, since our study was performed in a prospective cohort in a single institution and some patients admitted on Saturday were excluded due to our shortage of manpower. The performance of the at-point CFS as a universal risk indicator in acute inpatients should be confirmed in different populations or settings.

In conclusion, the at-point CFS assessed in older inpatients can screen high-risk individuals who may experience adverse geriatric conditions and hospital outcomes during their clinical course. This measure may serve as a universal risk indicator with the characteristics of a functional vital sign and it can be used to select an eligible population who may benefit from person-centered geriatric interventions in acute hospitals.

## Data Availability Statement

The raw data supporting the conclusions of this article will be made available by the authors, without undue reservation.

## Ethics Statement

The studies involving human participants were reviewed and approved by Institutional Review Board of Asan Medical Center. Written informed consent for participation was not required for this study in accordance with the national legislation and the institutional requirements.

## Author Contributions

H-WJ, JB, I-YJ, EL, and YK conceptualized the study and reviewed and edited the manuscript. H-WJ conducted the formal data analysis. H-WJ, JB, YhK, DK, H-SK, SL, HO, EL, and YK conducted the investigation. H-WJ and JB wrote the first draft of the manuscript. H-WJ, I-YJ, and YK supervised the study. All authors contributed to the article and approved the submitted version.

## Funding

This study was supported by a grant of the Korea Health Technology R&D Project through the Korea Health Industry Development Institute (KHIDI), funded by the Ministry of Health and Welfare, Republic of Korea (grant number: HI18C2383), and the Asan Multidisciplinary Committee for Seniors.

## Conflict of Interest

The authors declare that the research was conducted in the absence of any commercial or financial relationships that could be construed as a potential conflict of interest.

## Publisher's Note

All claims expressed in this article are solely those of the authors and do not necessarily represent those of their affiliated organizations, or those of the publisher, the editors and the reviewers. Any product that may be evaluated in this article, or claim that may be made by its manufacturer, is not guaranteed or endorsed by the publisher.
