# Supplementary material for: At-Point Clinical Frailty Scale as a Universal Risk Tool for Older Inpatients in Acute Hospital: A Cohort Study
Source: Front Med (Lausanne). 2022 Jul 6;9:929555. doi: 10.3389/fmed.2022.929555 (PMC9307996; doi:10.3389/fmed.2022.929555)
Supplement: Supplementary file 1 [file Data_Sheet_1.docx]

Supplementary Material

**Supplementary Table 1.** Sensitivities and specificities of Clinical Frailty Scale scores predicting in-hospital geriatric condition and at-risk population by risk scales.

|  | Fall incidence | | MFS≥45 |  | Pressure ulcer incidence | | BSS≤18 |  | Delirium incidence | |
| --- | --- | --- | --- | --- | --- | --- | --- | --- | --- | --- |
|  | Sen (%) | Spe (%) | Sen (%) | Spe (%) | Sen (%) | Spe (%) | Sen (%) | Spe (%) | Sen (%) | Spe (%) |
| ≥3 | 100.0 | 0.0 | 100.0 | 0.0 | 100.0 | 0.0 | 100.0 | 0.0 | 100.0 | 0.0 |
| ≥4 | 100.0 | 45.6 | 98.6 | 57.3 | 100.0 | 46.6 | 96.2 | 51.6 | 100.0 | 47.8 |
| ≥5 | 83.3 | 63.0 | 88.9 | 76.6 | 100.0 | 64.3 | 95.5 | 71.4 | 98.0 | 65.9 |
| ≥6 | 50.0 | 79.6 | 60.7 | 90.3 | 69.2 | 80.7 | 78.0 | 88.1 | 66.0 | 81.9 |
| ≥7 | 0.0 | 94.6 | 18.1 | 98.0 | 42.3 | 95.6 | 37.1 | 99.3 | 28.0 | 95.9 |
| ≥8 | 0.0 | 98.9 | 3.2 | 99.5 | 11.5 | 99.2 | 8.3 | 100.0 | 4.0 | 99.2 |

BSS, Braden sore scale; MFS, Morse fall scale; Sen, sensitivity; Spe, specificity

**Supplementary Table 2.** Sensitivities and specificities of the Clinical Frailty Scale scores predicting hospital outcomes.

|  | In-hospital death | | ED visit (30-day) | | Readmission (30-day) | | Composite outcome | |
| --- | --- | --- | --- | --- | --- | --- | --- | --- |
|  | Sen (%) | Spe (%) | Sen (%) | Spe (%) | Sen (%) | Spe (%) | Sen (%) | Spe (%) |
| ≥3 | 100.0 | 0.0 | 100.0 | 0.0 | 100.0 | 0.0 | 100.0 | 0.0 |
| ≥4 | 92.3 | 45.9 | 91.5 | 47.2 | 95.5 | 46.3 | 95.8 | 50.8 |
| ≥5 | 92.3 | 63.5 | 80.9 | 64.9 | 90.9 | 64.0 | 90.8 | 69.8 |
| ≥6 | 84.6 | 80.3 | 46.8 | 80.8 | 50.0 | 80.2 | 56.3 | 84.2 |
| ≥7 | 38.5 | 95.1 | 8.5 | 94.8 | 13.6 | 94.9 | 21.9 | 96.8 |
| ≥8 | 30.8 | 99.4 | 4.3 | 99.2 | 0.0 | 99.0 | 6.7 | 99.7 |

Sen, sensitivity; Spe, specificity

**Supplementary Table 3** List of the members of the Asan Multidisciplinary Committee for Seniors.

| Younsuck Koh, Department of Pulmonary and Critical Care Medicine, Health Screening and Promotion Center, Asan Medical Center  Soo-Sung Park, Department of Orthopedic Surgery, Asan Medical Center Children's Hospital, University of Ulsan College of Medicine  Eunju Lee, Division of Geriatrics, Department of Internal Medicine, Asan Medical Center, University of Ulsan College of Medicine  Dae Yul Kim, Department of Rehabilitation Medicine, Asan Medical Center, University of Ulsan College of Medicine  Hyouk-Soo Kwon, Department of Internal Medicine, Department of Allergy and Clinical Immunology, Asan Medical Center, University of Ulsan College of Medicine  Eun hee Cho, Innovation Designs Center, Asan Medical Center  Hye won Han, Pharmacy, Asan Medical Center  Sun hee Lee, Cancer Institute Nursting Team II, Department of Nursing, Asan Medical Center  Young gi Cho, Rehabilitation Medicine Team, Asan Medical Center  Jung sook Choi, Asan Medical Center Referral Center Team, Asan Medical Center  Hyun jin Oh, Surgical Nursing Team I, Department of Nursing, Asan Medical Center  Joo hyun Nam, Social Service Team,  Won gu Yi, Information Technology Service Team, Asan Medical Center  Ju yeon Oh, Information Technology Strategy Team, Asan Medical Center  Ki Young Son, Department of Family Medicine, Asan Medical Center, University of Ulsan College of Medicine  Hee-Won Jung, Division of Geriatrics, Department of Internal Medicine, Asan Medical Center, University of Ulsan College of Medicine |
| --- |
